# Acupuncture for the treatment of Delayed Encephalopathy after Acute Carbon Monoxide Poisoning: a case report

**DOI:** 10.3389/fmed.2026.1818476

**Published:** 2026-04-21

**Authors:** Jinlian Liao, Kaixuan He, Jijie Peng, Yan Wei, Yu Li, Yufeng He, Jiamin Wu

**Affiliations:** 1 Faculty of Chinese Medicine, Macau University of Science and Technology, Macau, China; 2 Zhongshan Hospital of Traditional Chinese Medicine, Zhongshan, China; 3 Zhuhai Hospital of Integrated Traditional Chinese and Western Medicine, Zhuhai, China

**Keywords:** _acupuncture therapy_, _cognitive impairment_, _Delayed Encephalopathy after Acute Carbon Monoxide Poisoning (DEACMP)_, _motor dysfunction_, _neurology_

## Abstract

**Background:**

Delayed Encephalopathy after Acute Carbon Monoxide Poisoning (DEACMP) is characterized by delayed neuropsychiatric deterioration after a period of apparent recovery. Effective treatments remain limited.

**Case presentation:**

A 75-year-old male developed cognitive decline, motor dysfunction, incontinence, and sleep disturbance persisting for more than 3 months after carbon monoxide poisoning despite standard therapy. MRI showed diffuse white matter demyelination.

**Intervention and outcomes:**

Zhu’s scalp acupuncture combined with body acupuncture was administered once daily for 15 sessions. Baseline MMSE and AD8 were not testable due to severe disorganized speech and poor cooperation. After treatment initiation, MMSE improved from 11 (after 1 session) to 14 (after 3 sessions), 24 (after 15 sessions), and 26 at one-month follow-up. AD8 decreased from 7 to 5, 2, and 1 at the corresponding time points. Functional independence was restored. No adverse events were reported.

**Conclusion:**

The temporal pattern of improvement suggests a possible association between acupuncture intervention and neurological recovery. Controlled studies are needed to further evaluate efficacy.

## 1 Introduction

Delayed Encephalopathy after Acute Carbon Monoxide Poisoning (DEACMP) is one of the serious complications of acute carbon monoxide poisoning. It is characterized by an insidious onset, a prolonged course, severely impacting the patient’s quality of life and placing a heavy burden on families and society (1). The core feature of this disease is that after successful resuscitation and recovery of consciousness, some patients may enter an asymptomatic or apparent recovery period lasting several days to several weeks. Neuropsychiatric symptoms may then emerge, including cognitive decline, behavioral changes, and motor dysfunction (2).

Currently, the pathogenesis of DEACMP is not fully understood, but it may be related to factors such as cerebral hypoxia, oxidative stress, demyelination of white matter, and neuronal apoptosis (3). Clinical treatment mainly focuses on using hyperbaric oxygen therapy to improve cerebral hypoxia and using neurotrophic drugs to protect neuronal cells (2). However, some patients do not show significant improvement in symptoms after the above treatments, highlighting the urgent need to explore more effective treatment strategies.

From a traditional Chinese medicine (TCM) perspective, Zhu’s scalp acupuncture is used to target functional disturbances of the brain by stimulating specific scalp acupoint lines (4), thereby providing a potentially useful adjunctive approach for the treatment of DEACMP. Here, we report a 75-year-old man with progressive cognitive decline, motor dysfunction, urinary/fecal incontinence, and sleep disturbance persisting for more than 3 months after CO poisoning despite standard therapy. At presentation, he was awake but fatigued and poorly cooperative, with marked impairment in memory, orientation, and calculation; limb strength was approximately 4/5 with no pathological reflexes. Brain imaging revealed diffuse cerebral white-matter demyelination, consistent with DEACMP.

## 2 A typical case

### 2.1 Case data

A 75-year-old male patient presented to our service with residual cognitive and motor dysfunction persisting for more than 3 months after recovery of consciousness from acute carbon monoxide (CO) poisoning. According to his caregiver, the patient experienced acute CO exposure during indoor charcoal heating in January 2025 and was treated at a local hospital with comprehensive management, including hyperbaric oxygen therapy and supportive/neurotrophic treatment; consciousness was restored after approximately 6 days. However, neurocognitive and functional deficits persisted and gradually progressed thereafter, manifested as an inability to answer daily questions normally and complete simple calculations. Concurrently, he had limb weakness and impaired voluntary urination and defecation (urinary and fecal incontinence). He also developed sleep disturbance, including difficulty initiating sleep and frequent nocturnal awakenings. These symptoms collectively led to a marked decline in functional independence, rendering him unable to perform activities of daily living independently.

On admission, he was awake but fatigued and poorly cooperative, with marked impairment in memory, orientation, and calculation. Pupils were equal and reactive, and cranial nerve screening was grossly unremarkable; motor strength was approximately 4/5 in all limbs with normal tone and reflexes, and Babinski signs were negative bilaterally; no meningeal signs were noted. Relevant past interventions included a brief trial of memantine and donepezil, as well as structured rehabilitation at a rehabilitation facility, with limited clinical improvement reported. Past medical history was notable for hypertension and coronary artery disease. He denied known drug/food allergies, smoking, alcohol abuse, or illicit drug use. Family history was unremarkable.

*Auxiliary examination*: cranial imaging revealed diffuse cerebral white matter demyelination, consistent with the imaging characteristics of DEACMP.

*Diagnosis*: delayed Encephalopathy after Acute Carbon Monoxide Poisoning (DEACMP).

*Diagnostic assessment and challenges*: differential diagnoses included vascular cognitive impairment related to cerebrovascular stenosis/lacunar infarctions, Alzheimer’s disease, and other causes of encephalopathy. However, the temporal relationship to CO poisoning, multi-domain neuropsychiatric deterioration, and diffuse white-matter involvement supported a diagnosis of DEACMP. The precise duration of the asymptomatic/recovery interval could not be reliably determined because early symptom evolution was reconstructed from caregiver report and prior medical records were incomplete.

*TCM syndrome differentiation*: invasion of toxic pathogens, phlegm and blood stasis obstructing the clear orifices. Due to the invasion of toxic pathogens, which stagnate and transform into heat, consuming body fluids to form phlegm, phlegm and blood stasis mutually accumulate, blocking the meridians and obscuring the clear orifices, leading to cerebral dysfunction. Consequently, cognitive, motor, and urinary/fecal dysfunctions occur. Therapeutic principle: resuscitating the brain, opening the orifices, and dredging the meridians.

### 2.2 Treatment plan

#### 2.2.1 Selection of needles

Disposable sterile acupuncture needles (0.25 mm × 20 mm) conforming to the national standard “Disposable Sterile Acupuncture Needles” were employed.

#### 2.2.2 Acupoint selection

Main acupoints (Zhu’s Scalp Acupuncture): Bilateral Anterior Temporal Zone (Nieqian Dai, corresponding to the motor area) and Posterior Temporal Zone (Niehou Dai, corresponding to the sensory area).

Body acupoints (Body Acupuncture): Neiguan (PC6), Hegu (LI4), Taichong (LR3), Fenglong (ST40), Renzhong (GV26), Sanyinjiao (SP6).

**Figure 1:**
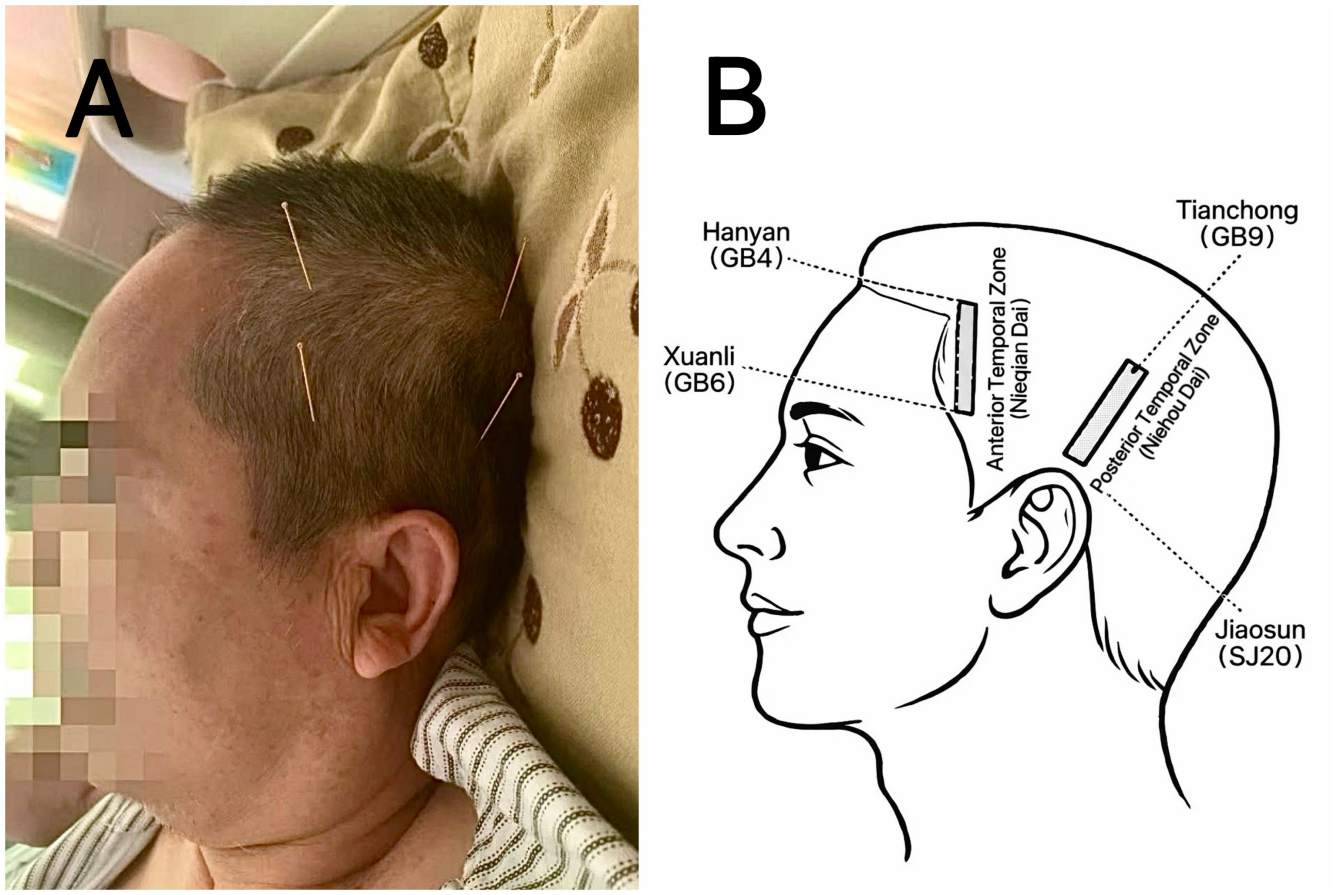
Acupoint localization and clinical procedure of Zhu’s scalp acupuncture. **(A)** Representative clinical images demonstrating needle insertion and manipulation during treatment. **(B)** Schematic diagram showing the bilateral anterior temporal zone (Nieqian Dai) and posterior temporal zone (Niehou Dai), redrawn by the authors with reference to Zhu and Peng (15).

See Figure 1 for acupoint localization and the clinical procedure.

#### 2.2.3 Operation methods

Scalp Acupuncture Operation: the patient was placed in a supine position. After routine disinfection of the scalp, the needle was inserted horizontally along the selected acupoint line at a 30° angle to the scalp, rapidly penetrating the subgaleal space until deqi (needling sensation) was achieved. Subsequently, high-frequency and small-amplitude twisting manipulation was performed for 1–2 min (twisting frequency: approximately 120–160 cycles per minute). The needle was retained for 30 min, and manipulation was repeated every 10 min to enhance stimulation.

Body Acupuncture Operation: after routine disinfection of the acupoint skin, lifting-thrusting and twisting reinforcing-reducing manipulations were administered according to the characteristics of the acupoints and the patient’s syndrome. Among them, reducing manipulation was applied at Neiguan (PC6) and Renzhong (GV26) to induce resuscitation and dredge the meridians; reinforcing manipulation was used at Sanyinjiao (SP6) to nourish the kidney and marrow, and replenish qi and blood; even reinforcing-even reducing manipulation was adopted at Hegu (LI4), Taichong (LR3), and Fenglong (ST40) to regulate qi and blood, resolve phlegm, and dredge the meridians.

Adjunctive Interventions: during needle retention, under the guidance of medical staff, passive limb movements (e.g., joint flexion and extension, muscle massage) and active limb function exercises (e.g., limb lifting, grip strength training) were performed to implement the concept of “retaining the needle with movement” and promote the remodeling of neural pathways.

All acupuncture sessions were performed by the same licensed acupuncturist, who had clinical experience in Zhu’s scalp acupuncture. The treatment protocol, including acupoint selection, insertion angle, needle retention time, and manipulation frequency, was kept as consistent as possible across sessions. Deqi was sought at both scalp and body acupoints whenever feasible and tolerated by the patient. No major changes were made to the acupoint prescription during the treatment course.

#### 2.2.4 Course of treatment

Treatment was administered once daily, with 15 consecutive sessions constituting one course. Outcomes were assessed after completion of the course and again at a one-month follow-up.

## 3 Efficacy observation

### 3.1 Evaluation criteria

Cognitive function was assessed using the Mini-Mental State Examination (MMSE) (total score: 30 points; higher scores indicate better cognitive function) (5); dementia-related behavioral changes were assessed using the AD8 Dementia Screening Interview (AD8) (total score: 8 points; higher scores indicate more severe functional impairment) (6).

### 3.2 Efficacy results

The episode of care is summarized as follows: acute CO poisoning in January 2025 treated with hyperbaric oxygen therapy; persistent neurocognitive decline and functional impairment despite rehabilitation; initiation of acupuncture at >3 months post-exposure with serial MMSE and AD8 follow-up as shown in Table 1.

**Table 1:** Timeline of clinical course, interventions, and outcomes.

Time point	MMSE (0–30)	AD8 (0–8)	Cognitive status	Motor function	Autonomic function	Functional independence
Pre-treatment	Not testable	Not testable	Severe cognitive impairment; unresponsive to questioning; slurred speech	Generalized limb weakness	Urinary and fecal incontinence	Fully dependent
After 1 treatment	11	7	Minimal improvement; able to follow simple commands	Slight improvement in limb weakness	No significant change	Dependent
After 3 treatments	14	5	Able to provide brief responses to daily questions	Improved limb muscle strength	No significant change	Partial assistance required
After 15 treatments	24	2	Able to answer questions normally and perform basic calculations	Able to stand and walk independently	Continence restored	Independent
1-month follow-up	26	1	Stable cognition; improved memory and orientation	Stable motor function	Normal	Independent

The table presents the treatment timeline and serial outcomes following acupuncture initiation; key pre-treatment events are summarized in the Results section.

At pre-treatment assessment, MMSE and AD8 were not testable due to severe disorganized speech and poor cooperation. After initiation of acupuncture, MMSE increased from 11 (after 1 session) to 14 (after 3 sessions), 24 (after 15 sessions) and 26 at one-month follow-up, while AD8 decreased from 7 to 5, 2, and 1 at the corresponding time points. These quantitative improvements were accompanied by progressive recovery of motor and autonomic function, and the patient regained independence in activities of daily living by the end of the treatment course. Cognitive and functional status remained stable at follow-up. No adverse events were observed. The patient completed all planned sessions without missed treatments.

## 4 Discussion

In this case, objective and progressive improvement in cognitive and functional measures was observed during the treatment period. MMSE scores increased from 11 after the first session to 24 at completion of one treatment course and further to 26 at one-month follow-up, while AD8 scores declined from 7 to 1. These quantitative changes were accompanied by recovery of independent ambulation, restoration of continence, and regained autonomy in daily activities. It should be acknowledged that spontaneous recovery may occur in the early phase of DEACMP. However, the stepwise and temporally aligned improvements observed during the intervention period, together with parallel recovery across multiple functional domains, provide clinical observations of interest. While causality cannot be established, the temporal association between treatment and recovery warrants further systematic investigation.

In the clinical context of Delayed Encephalopathy after Acute Carbon Monoxide Poisoning (DEACMP), such recovery is clinically noteworthy. DEACMP often develops weeks to months after the acute insult, and persistent cognitive and motor deficits may remain despite standard treatment, including hyperbaric oxygen therapy (2). Therefore, identifying interventions that may facilitate neurological recovery during the subacute stage is of clinical interest.

Several mechanisms may underlie the improvement observed in this case, although they remain speculative. Firstly, by stimulating the acupoint lines corresponding to the motor and sensory areas of the scalp, it may help promote the establishment of cerebral collateral circulation, improve the hypoxic state of the cerebral cortex, and provide a favorable microenvironment for the repair of damaged neuronal cells (7, 8). Secondly, it may modulate the balance of oxidative stress in the body, increase the activity of superoxide dismutase (SOD), reduce the level of lipid peroxidation (LPO), and mitigate free radical-induced damage to neuronal cells (9, 10). Thirdly, based on the TCM theories that “the brain is the residence of the primordial spirit” and “meridians traverse corresponding regions, governing their disorders,” it may facilitate cerebral meridians, activate cerebral function, and potentially facilitate the remodeling and repair of damaged neural pathways (11). However, these possible mechanisms were not directly examined in the present case and should be interpreted cautiously. In addition, recent studies have highlighted the importance of meningeal and cervical lymphatic drainage in brain waste clearance and neurodegenerative disease, and some preclinical studies have suggested that acupuncture-related interventions may influence glymphatic- or lymphatic-related clearance pathways (12–14). These findings provide an additional perspective for understanding the potential mechanisms of acupuncture in neurological recovery. Although lymphatic or glymphatic function was not assessed in the present case, this pathway may merit further investigation in future studies of DEACMP.

The key factors that may have contributed to the observed improvement in this case are as follows:

① Early intervention: the patient was still possibly in the window of neurological function repair 3 months after the onset of the disease, and the timely application of Zhu’s scalp acupuncture may have laid the foundation for the treatment effect.

② Precise acupoint selection: the anterior and posterior nerve acupoints correspond to the motor and sensory areas respectively, which may help improve motor and sensory functions. Combined with Neiguan (PC6) and Renzhong (GV26) to activate the brain and open the orifices, Fenglong (ST40) to resolve phlegm and unblock the meridians, and Sanyinjiao (SP6) to nourish the kidney marrow, a comprehensive acupoint selection system targeting the etiology and symptoms was formed.

③ Standardized manipulation: high-frequency and small-amplitude twisting of scalp acupuncture, syndrome differentiation-based reinforcing-reducing of body acupuncture, combined with the concept of “retaining the needle with movement”, may have realized the “integration of acupuncture and movement”, thereby potentially accelerating the recovery of neurological function.

Nevertheless, several limitations should be acknowledged. This report describes a single case, and causal inference cannot be established. Although the temporal pattern of improvement suggests a possible association with the intervention, spontaneous recovery in DEACMP cannot be entirely excluded, particularly within the early months after injury. In addition, no follow-up MRI or biomarker assessments were performed to evaluate structural or biochemical changes objectively. These factors limit the generalizability of the findings.

Future studies incorporating larger cohorts and controlled designs are needed to further evaluate the therapeutic potential of Zhu’s scalp acupuncture combined with body acupuncture in DEACMP. Integration of longitudinal neuroimaging, electrophysiological monitoring, and oxidative stress biomarkers may help clarify underlying mechanisms and identify patients most likely to benefit. Such approaches would strengthen the evidence base and contribute to a more comprehensive understanding of its role in neurorehabilitation.

## 5 Conclusion

In this case, Zhu’s scalp acupuncture combined with body acupuncture was temporally associated with progressive improvement in cognitive, motor, and autonomic function in a patient with Delayed Encephalopathy after Acute Carbon Monoxide Poisoning (DEACMP). The intervention was well tolerated and feasible within a clinical rehabilitation setting. Given the inherent limitations of a single-case report, the findings should be interpreted with caution. Controlled studies with larger cohorts, along with longitudinal imaging and mechanistic investigations, are required to further clarify therapeutic efficacy and underlying biological mechanisms.

## 6 Patient perspective

At the beginning of treatment, the patient was unable to provide a reliable self-report due to severe cognitive impairment and disorganized speech. According to his family, before acupuncture he could not communicate normally, wandered at night, and required full assistance for daily activities, which placed a substantial caregiving burden. During the treatment course, the family observed progressively clearer responses and improved daily function. At follow-up, the patient was able to answer questions appropriately and reported feeling “more clear-headed” and more confident in daily activities.

## Data Availability

All data generated or analyzed during this study are included in this published article. Given the single-patient design of this case report and the need to protect patient privacy, no additional data are publicly available. Requests for further information should be directed to the corresponding author and will be considered in accordance with institutional ethical regulations.
